# Nanowire CdS-CdTe Solar Cells with Molybdenum Oxide as Contact

**DOI:** 10.1038/srep14859

**Published:** 2015-10-06

**Authors:** Hongmei Dang, Vijay P. Singh

**Affiliations:** 1 Department of Electrical and Computer Engineering, and Center for Nanoscale Science and Engineering (CeNSE), University of Kentucky, Lexington, KY, 40506-0046, USA

## Abstract

Using a 10 nm thick molybdenum oxide (MoO_3−x_) layer as a transparent and low barrier contact to p-CdTe, we demonstrate nanowire CdS-CdTe solar cells with a power conversion efficiency of 11% under front side illumination. Annealing the as-deposited MoO_3_ film in N_2_ resulted in a reduction of the cell’s series resistance, from 9.97 Ω/cm^2^ to 7.69 Ω/cm^2^, and increase in efficiency from 9.9% to 11%. Under illumination from the back, the MoO_3−x_/Au side, the nanowire solar cells yielded J_sc_ of 21 mA/cm^2^ and efficiency of 8.67%. Our results demonstrate use of a thin layer transition metal oxide as a potential way for a transparent back contact to nanowire CdS-CdTe solar cells. This work has implications toward enabling a novel superstrate structure nanowire CdS-CdTe solar cell on Al foil substrate by a low cost roll-to roll fabrication process.

CdTe^1^, Cu(In,Ga)Se_2_^2^, Cu_*2*_ZnSn(S,Se)_4_^3^, silicon^4^, and perovskites^5^ are among the leading photovoltaic technologies being developed to generate low-cost solar electricity. In particular, CdTe photovoltaics have energy return investment exceeding that of traditional fossil fuels and provide the shortest energy payback time among all photovoltaic technologies for terrestrial applications^6^. In addition, CdTe photovoltaics have superior tolerance to high energy irradiation and are more suitable for space applications^7^.

Development of a transparent and stable contact to the CdTe absorber layer has remained challenging and is of great interest because it can further advance the technology where CdTe solar cells are fabricated on flexible foils of metals in a superstrate device structure. The metal foil-based CdTe solar cells can be implemented by a high-throughput roll-to-roll manufacturing process, resulting in significant cost reduction, high material utilization and fabrication scalability^7^. Traditionally, CdTe solar cells on metal foils are configured with an inverted substrate structure^6^,^7^. However, CdTe solar cells using the substrate structure yield lower efficiency values than their superstrate counterparts^7^. The substrate structure imposes many restriction on process optimization, for example, the rather difficult etching process on the CdTe layer prior to contact formation, diffusion of impurities to the contact of CdTe, and CdCl_2_ treatment effect on CdS and CdTe layer^6^,^7^. Hence, formation of superstrate structured CdTe solar cells on metal foils is one of the most promising options for low cost and high efficiency photovoltaic technologies.

Recently, we have developed nanowire CdS-CdTe solar cells to address light absorption loss and interface recombination issues^8^,^9^,^10^,^11^ , where CdS nanowires embedded in a transparent anodic aluminum oxide (AAO) membrane replace planar CdS as the window layer and CdTe is deposited on the top of the CdS nanowires (Fig. 1a)^12^. Such nanowire CdS-CdTe solar cells reduced light absorption loss in the low wavelength region by confinement effects of nanostructures^8^,^9^,^10^,^11^, and exhibited a nearly ideal spectral response of quantum efficiency from 335 nm to 850 nm, which is the cut-off wavelength corresponding to the bandgap of the CdTe absorber. Next, it was thought that the development of a transparent back contact for CdTe film would lead to a nanowire CdS-CdTe solar cells that would allow the shining of sunlight through the back contact and thus realize the superstrate configuration (Fig. 1b). Because AAO is formed by anodizing aluminum, the nanowire CdS-CdTe solar cells can be grown on aluminum foil; this makes the roll-to-roll manufacturing process feasible and greatly reduces the complexity of fabrication (Fig. 1c).

Several types of metal oxides have been investigated as buffer layer to low barrier back contacts^13^. In this work, molybdenum oxide (MoO_3_) is studied as a transparent back contact to the CdTe absorber layer, due to its high transparency (higher than 80%) in the visible and near IR range and its behavior is like that of a high work function metal^14^,^15^,^16^. In addition, its electrical and optical properties can be tuned by controlling the oxygen stoichiometry during processing. Several groups have applied MoO_3_/Ni and MoO_3_/Au back contacts into CdS/CdTe solar cells and reported 12.2–14.1% values for power conversion efficiency^17^,^18^,^19^. Here, we investigate the effects of a thin MoO_3_/Au layer as the transparent back contact on the nanowire solar cells through front and back side illuminations. We further investigate possibility of reducing resistance of MoO_3_ back contacts due to its insulation property by post-processing annealing. In the following sections, the effects of the MoO_3_/Au back contact layer on the structural and device properties of the nanowire CdS-CdTe solar cells are demonstrated and their loss mechanism and further improvement are discussed.

## Experimental procedures

The nanowire CdS/CdTe solar cells are prepared by our previously established methods^12^. The solar cells were fabricated on ITO coated soda-lime glass substrates with sheet resistance of 23–28 Ω/square. The fabrication processes include formation of AAO membrane by anodizing aluminum film, electro-deposition of CdS nanowires with 100 nm height, close-space sublimation of CdTe to a thickness of 10 μm, and CdCl_2_ treatments at 400 °C. Without NP etch, MoO_3_ thin films with a thickness of 10 nm were thermally evaporated on clean CdTe surfaces from stoichiometric MoO_3_ powder (Alfa Aesar, 99.9%), where the pressure was less than 1*10^−5^ Torr and the deposition rate was maintained as 0.5 Å/s. Samples were masked and then annealed at 200 °C in N_2_ for 10 mins. In the last step, 15 nm Au was deposited by sputtering process. For a comparative study, after thermal evaporation of molybdenum oxide, samples were directly coated with 15 nm of Au without the intervening annealing step. After depositing Au layer, all of the samples were annealed at 200 °C in Argon for 10 mins. Structures of CdS nanowires embedded in AAO templates were characterized via scanning electron microscopy (S-900-SEM). Current–voltage (I –V) was measured by a solar simulator set at 100 mW/cm^2^, calibrated by a power meter.

## Results and Discussion

### Materials Characterization

**Figure 2:**
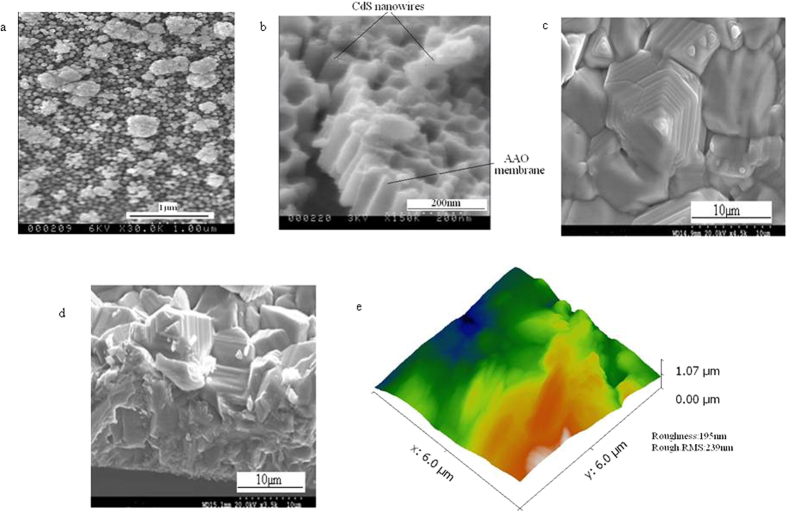
(**a**) Top view SEM image of free-standing CdS nanowires, (**b**) Cross-sectional view SEM images of CdS nanowires embedded in AAO membrane, (**c**) Top view SEM image of CdTe film, (**d**) cross-section view of CdTe film. (**e**) The AFM image of the top view of CdTe.

CdS nanwires are characterized by scanning electron microscopy (SEM). Figure 2a shows free standing CdS nanowires where AAO membrane has been completely removed by a highly selective NaOH solution. Figure 2b shows cross-sectional view of CdS nanowires. As seen in Fig. 2b, CdS nanowires are embedded in AAO nanopores; often, a few nanopores are seen to be missing their CdS nanowires; these, very likely, were knocked off from the AAO nanopores during the sample preparation steps for the SEM viewing. The sample preparation for cross section requires scoring the film hard so that from the shock, a fracture exposing the cross section is achieved.

On the other hand, in the SEM sample preparation for the top view (Fig. 2a), no such fracturing shock is required. In the top views of our films, all the AAO pores are in fact filled by the CdS nanowires.

Overall, CdS nanowires form uniform and dense arrays; values of the average length of nanowires, the average diameter and the average distance between the centers of neighboring nanowires are 100 nm, 60 nm and 106 nm respectively. Based on the CdS nanowires features, porosity is approximately 32% and area density of CdS nanowires is calculated as approximately 1.14*10^10^ nanowires/cm^2^. This high density CdS nanowire array was grown perpendicular to the glass-ITO substrate, but can also be directly grown on aluminum foil and other flexible substrates. These embedded CdS nanowires function as the window layer and are configured into the CdS nanowire window layer-CdTe absorber layer solar cells.

Figure 2c,d show the surface morphologies of CdTe film and cross-section view of CdTe film. It is obvious that CdTe film have a compact morphology, consisting of crystallites with grain size between average 6 μm to 7 μm. Observed from cross section SEM images of the CdTe film, it is estimated that thickness of CdTe is the 10 μm–15 μm. Figure 2e shows the AFM image of the top view of CdTe. For 6*6 μm^2^ CdTe area, the roughness and roughness rms values are 195 nm and 239 nm.

### Quantum Efficiency of the Nanowire CdS-CdTe Solar Cells

**Figure 3:**
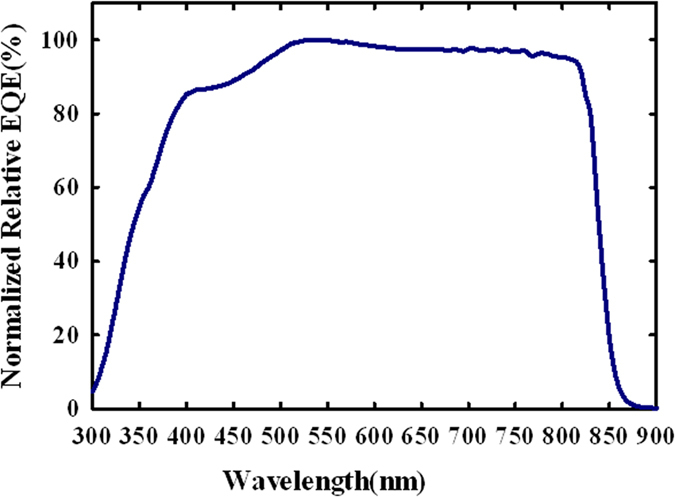
Normalized relative EQE of the nanowire CdS/CdTe solar cells on intrinsic SnO_2_/ITO/Soda-lime glass substrate.

Quantum efficiency characterization is especially interesting in exploring how the CdS nanowires embedded in the AAO membrane as the window layer effectively improve light transmission and how the nanowire CdS-CdTe solar cells effectively absorb light and generate and collect carriers by confinement effects of nanostructures^8^,^9^,^10^,^11^. The normalized external quantum efficiency (EQE) of a typical nanowire solar cell is shown in Fig. 3. These nanowire solar cells were fabricated on intrinsic SnO_2_/commercially available ITO-soda lime glass substrate with low transparency and high resistivity.

As shown in Fig. 3, the nanowire CdS-CdTe solar cells exhibit relatively strong quantum efficiency response from 345 nm to 845 nm, which is the bandgap edge of CdTe absorber. Such EQE response indicates that a very wide spectral range of incident photons is almost completely absorbed and photogenerated carriers are effectively collected. It is clear that CdS nanowires embedded in transparent AAO membrane effectively enhance transmission of the window layer. As a result, the wide spectrum of sunlight above 345 nm can be directed into the CdTe absorber where photons are absorbed and converted into charge carriers. Thus by using the embedded CdS nanowires as the window layer, abilities of carrier generation and collection in the CdS-CdTe solar cells are effectively enhanced.

### Photovoltaic Characteristics of Nanowire CdS-CdTe-MoO_3−x_-Au solar cells

**Figure 4:**
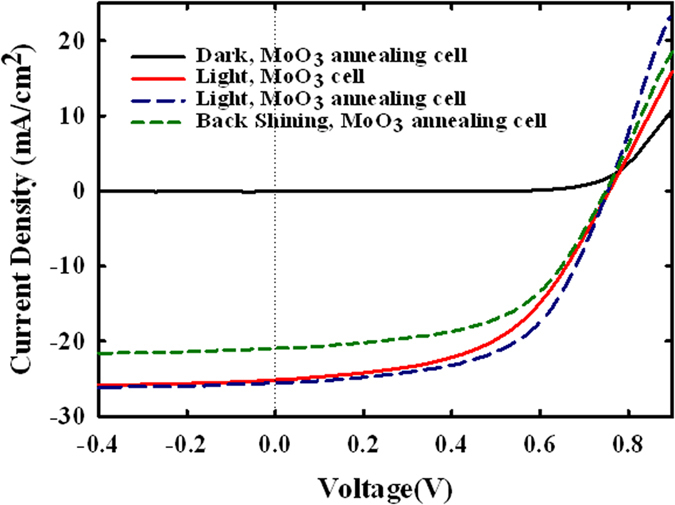
J-V curves of nanowire CdS-CdTe solar cells with as deposited MoO_3_/Au back contacts under front side illumination, annealing MoO_3_/Au back contacts under dark, front side and back side illuminations.

The performance of the nanowire CdS-CdTe solar cells with MoO_3_/Au back contacts was characterized. Figure 4 shows the J-V characteristics of the nanowire solar cells with MoO_3_/Au contacts with N_2_ annealing under dark, 1-Sun front side illumination and 1-Sun back side illumination, as well as the cells with MoO_3_/Au contacts without N_2_ annealing under 1-Sun front side illumination. There is no roll-over effect observed in any of the light J-V curves. Hence, incorporation of MoO_3_/Au as a back contact to CdTe film eliminates the commonly observed blockage to hole transport across the interface between the CdTe layer and the “traditional” back contact. It has been reported that MoO_3_ has high work function of 5.5 eV–6.86 eV and has a behavior like that of a metal^15^,^16^. This high work function of MoO_3_ is thought to form a well-aligned buffer layer and to reduce the effective barrier height, thus facilitating the formation of quasi- ohmic back contacts to CdTe.

The optimal thickness of the MoO_3_ layer is observed to be in the 5 nm–10 nm range., while the thickness of CdTe by closed-space sublimation is about 10 μm–12 μm. Thinner MoO_3_ layer, less than 5 nm, is not enough to guarantee a continuous coverage and efficient contacts for hole transport due to surface roughness of CdTe. Thicker MoO_3_ layer, more than 10 nm, leads to decrease of short current density (J_sc_) and fill factor (FF) due to high resistivity of the MoO_3_ layer. To reduce resistance and guarantee a continuous layer, we deposited the 10 nm MoO_3_ layer as the buffer layer of back contacts for CdTe absorber in this work. Table 1 summarizes cell performance parameters.

The 195 nm roughness value of the surface of the CdTe absorber layer is small enough that a conformal covering of the MoO_x_ electrode layer over CdTe is obtained when the thickness of MoO_x_ is more than 5 nm. When the thickness of the MoO_x_ buffer layer was smaller than 5 nm, the current-voltage characteristics of the completed NW-CdS/CdTe cell exhibited the “roll-over effect”, which is associated with a non-conducting, Schottky diode behavior at the CdTe-eletrode interface. This would be expected to happen when the CdTe was not fully covered by the MoO_x_ buffer layer; then, there would be patches of CdTe surface in direct contact with the Au electrode, which forms a Schottky diode with p-CdTe. To avoid this undesirable outcome, in our experiments, the thickness of the MoOx buffer layer was set at 10 nm.

The reported cell performance was achieved on intrinsic SnO_2_/ITO-soda-lime glass substrates and without antireflective coating.

The new Table 1 lists the variability range for each parameter for a statistical sample of 3; the average value for each parameter is listed in the new Table 2. Comparing the first two rows of the Efficiency column in these Tables, it is seen that the annealing step improved the average efficiency value by 1.03% (from 9.97% to 11%); this increase is much larger than the variability range values of 0.23% and 0.22% and, therefore, of practical significance.

Similarly, comparing the last two rows of the Efficiency column in these Tables, it is seen that under back side illumination, the average efficiency value decreased by 2.33% (from 11% to 8.67%); this reduction is much larger in magnitude than the variability range values of 0.29% and 0.22% and, therefore, of practical significance.

As comparison, these solar cell parameter values are lower than the V_oc_ of 770 mV, J_sc_ of 26 mA/cm^2^, fill factor of 60% s, and power conversion efficiency of 12% seen in the best nanowire CdS-CdTe solar cell with Cu/graphite back contacts^18^,^19^. Hence there is room for further optimization and performance improvement. Low shunt resistance might have been caused by contribution from the incomplete isolation of cells and less than satisfactory scribing of intrinsic SnO_2_. Lower fill factor and J_sc_ are attributed to high series resistance, which is higher than the series resistance of planar CdS-CdTe solar cells. Fully stoichiometric MoO_3_ with only Mo^6+^ is insulating and has a rather high resistivity of 10^3^–10^4^ Ωcm^18^,^19^. Although J-V curves show that the incorporation of thin MoO_3_ layer does not lead to the roll-over behavior, still, high resistivity attribute of MoO_3_ may lead to some blockage of hole transport. This would show up as a high effective series resistance, leading to lower values fill factor and J_sc_.

When MoO_3_ is exposed to N_2_ annealing before depositing Au as back contact, the performance of the nanowire solar cells is improved. The average J_sc_ and fill factor are improved to 25.6 mA/cm^2^ and 57.1% respectively, and the average series resistance is reduced from 9.97 Ω/cm^2^ to 7.69 Ω/cm^2^, and shunt resistance is increased to 332.2 Ω/cm^2^, leading to power conversion efficiency of approximately 11%. The measured error of the efficiency is 1%. It has been reported earlier that after annealing in N_2_, a small amount of MoO_2_ as well as Mo^4+^ ions are present in the MoO_3_ film, and MoO_2_ is metallic^15^,^16^. Consequently, it is thought that after annealing at 200 °C in N_2_ for 10 minutes, evaporated MoO_3_ film has in it the mixed oxidation states of Mo, mainly attributed to MoO_3_ and MoO_2_ phases^14^,^15^,^16^. These mixed oxidation states of Mo (MoO_3−x_) can sustain the dominant high work function behavior arising from MoO_3_ and also reduce resistivity due to metallic behavior of MoO_2_ phase. Hence, series resistance of the nanowire solar cells is significantly reduced, and fill factor and power conversion efficiency are improved. An increase in J_sc_ for the annealing MoO_3_ (MoO_3−x_) back contact case is attributed to reduced barrier height due to lower resistivity of MoO_3−x_/Au back contacts.

To illustrate the feasibility of a relatively transparent MoO_3−x_/Au hole selective contact to p-CdTe, we illuminated the nanowire solar cells from back-contact side (MoO_3−x_/Au side rather than SnO_2_/ITO/Soda-lime glass side). The resulting J-V characteristics and the photovoltaic performance are shown in Fig. 4 and Table 1. Under back side illumination, the nanowire CdS-CdTe solar cell with MoO_3−x_/Au back contact exhibits average J_sc_ of 21 mA/cm^2^, V_oc_ of 749 mV, fill factor of 55.1%, corresponding to an average power conversion efficiency of 8.67%; this is much higher than the 5.8% value reported for the nanopillor CdS-CdTe solar cells with Cu/Au (1 nm/13 nm) back contacts from back side illumination^20^.

When comparing with front-side illumination, thicker MoO_3−x_ (10 nm) and Au (15 nm) are responsible for low optical transmission. Although MoO_3−x_ has bandgap of 3.0–3.8 eV and high transparency of more than 80% from 400 nm to near IR range, still, Au of 15 nm thickness could cause relatively strong transmission losses. Hence, obvious decrease in efficiency is on account of lower J_sc_ based on low transmission from back side illumination. By further exploring transparent back contacts of CdTe, for example, transparent metal at nanoscale in the future, the superstrate structured nanowire CdS-CdTe solar cells on Al substrate can become a low complexity and high efficiency solar technique.

**Figure 5:**
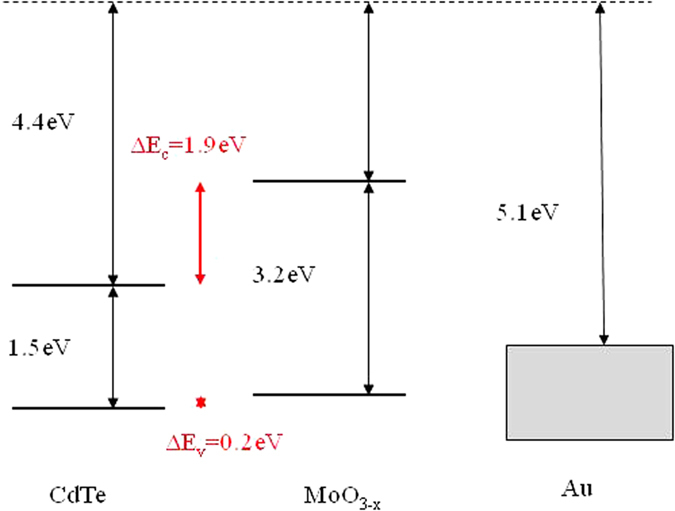
Energy Band Discontinuities between the CdTe absorber and the MoO_3−x_/Au back contact layers.

Considering that MoO_3−x_ behaves like a high work function metal with a low density of states at the Fermi level and has transparent properties in the visible and near IR range^14^,^15^, it is chosen as a transparent back contact candidate for nanowire CdS-CdTe solar cells. For a better understanding of the MoO_3−x_ back contacts, the energy band diagram of the junction between CdTe and MoO_3−x_/Au is illustrated in Fig. 5 below. A work function of 5.7 eV and energy bandgap of 3.2 eV are assumed for MoO_3−x_, and the electron affinity of 4.4 eV and energy band of 1.5 eV are assumed for CdTe^14^. Figure 5 illustrates the energy band discontinuities between CdTe and MoO_3−x_/Au . It is noted that Au, when placed directly next to p-CdTe, would form a blocking, Schottky diode contact with a barrier height of 0.8 eV. This would prevent hole transport from CdTe to Au contact and reduce cell performance. Introducing the thin MoO_3−x_ interlayer between CdTe and Au removes the Schottky diode problem. Now, a valance band offset of approximately 0.2 eV occurs between the CdTe and the MoO_3−x_ layers. Thus, MoO_3−x_ layer functions as a well-aligned buffer layer to reduce barrier height relative to CdTe. Hence it plays an important role to ensure hole extraction and transport to the electrode. As a result, the nanowire CdS-CdTe solar cells yield enhanced performance. In addition, due to its transparent properties, MoO_3−x_ layer, as a back contact to CdTe provides a potency to achieve superstrate structured nanowire solar cells on flexible metal foil substrate.

Our nanowire CdS-CdTe cell design with MoO_3−x_/Au back contacts has demonstrated the performance comparable to that of planar counterpart with MoO_3−x_/metal back contacts under front side illumination^17^,^18^,^19^. For back side illumination, it has exhibited performance much better than that of nanopillar CdS-CdTe solar cells with Cu/Au contact^20^, and its performance is comparable to that of planar CdS-CdTe solar cells with substrate structure^7^,^21^. It clearly illustrates the concept of MoO_3−x_ as a transparent hole selective contact to p-CdTe due to its well-aligned band structure.

### Conclusions and Future Work

We have fabricated nanowire CdS-CdTe solar cells and introduced MoO_3−x_ as a transparent, low barrier back contact. The MoO_3_ layer reduces the valence band offset relative to the CdTe, and creates improved cell performance. Annealing as-deposited MoO_3_ in N_2_ reduces series resistance from 9.97 Ω/cm^2^ to 7.69 Ω/cm^2^, and hence efficiency of the nanowire solar cell is improved from 9.9% to 11%. When the nanowire solar cell is illuminated from MoO_3−x_/Au side, it yields an efficiency of 8.67%. This reduction in efficiency is attributed to decrease in J_sc_ from 25.6 mA/cm^2^ to 21 mA/cm^2^ due to light transmission loss in the MoO_3−x_/Au electrode. Even though these nanowire solar cells, when illuminated from back side exhibit better performance than that of nanopillar CdS-CdTe solar cells structure^7^,^21^, further development of transparent back contacts of CdTe could enable a low-cost roll-to-roll fabrication process for the superstrate structure-nanowire solar cells on Al foil substrate.

Various potential improvements of our nanowire solar cell design with MoO_3−x_/metal back contacts can be envisioned including optimization of MoO_3−x_ layer to further reduce resistance, and optimization of CdS nanowires and CdTe layer to further improve V_oc_ and J_sc_. The FF could be improved by replacement or ideal scribing of intrinsic SnO_2_ to increase shunt resistance and optimization of MoO_3−x_ layer for low series resistance. Development of a transparent metal layer on the MoO_3−x_ will improve light transmission loss and significantly enhance J_sc_ under back-side illumination. Furthermore, MoO_3−x_ with the transparent metal as the back contacts of CdTe could make the nanowire solar cells on Al foil with superstrate structure promising and facilitate a roll-to-roll fabrication process application on such solar cells, thus providing a route toward a scalable, low-cost solar cell architecture.

## Additional Information

**How to cite this article**: Dang, H. and Singh, V. P. Nanowire CdS-CdTe Solar Cells with Molybdenum Oxide as Contact. *Sci. Rep.* **5**, 14859; doi: 10.1038/srep14859 (2015).

## Figures and Tables

**Figure 1:**
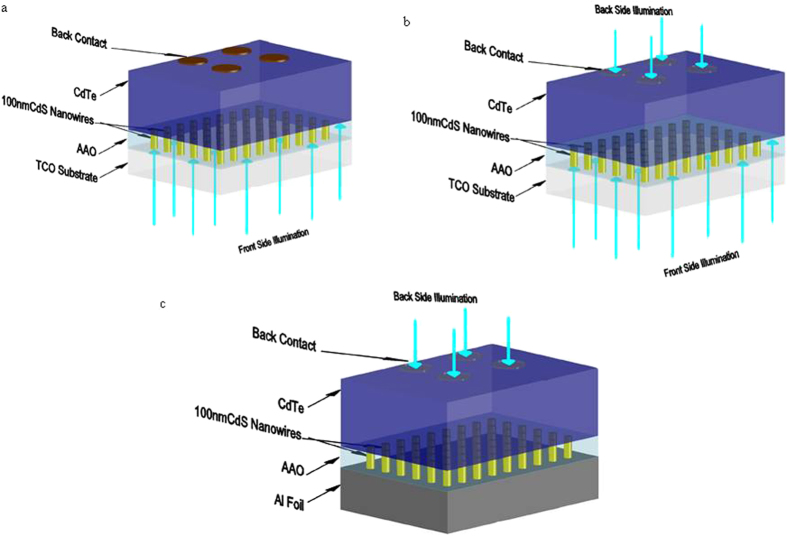
(**a**) Schematic of a CdS nanowire window layer-CdTe absorber solar cell on TCO substrate illuminated from front side. (**b**) Schematic of a CdS nanowire window layer-CdTe absorber solar cell on TCO substrate with almost transparent back contacts which can be illuminated from front and back side. (**c**) Schematic of a CdS nanowire window layer-CdTe absorber solar cell on Al foil with almost transparent back contacts which can be illuminated from back side.

**Table 1:** Photovoltaic properties of nanowire CdS-CdTe solar cells with MoO_3_/Au as back contacts (Ranges of Variability).

Back Contacts	Illumination Conditions	J_sc_(mA/cm^2^)	V_oc_ (mV)	FF(%)	Efficiency (%)	Rs(Ω/cm^2^)	Rsh(Ω/cm^2^)
MoO_3_/Au	Front-Side	24.8–25.4	755–756	52–52.2	9.77–10	9.8–10.13	300–308
MoO_3_/Au annealing	Front-Side	25.4–25.8	752–753	56.9–57.2	10.9–11.12	7.58–7.78	328–336
MoO_3_/Au annealing	Back-Side	20.7–21.4	749–750	55.1–55.2	8.55–8.84	9.93–10.35	338–350

**Table 2:** Photovoltaic properties of nanowire CdS-CdTe solar cells with MoO_3_/Au back contacts (Average Values).

Back Contacts	Illumination Conditions	J_sc_(mA/cm^2^)	V_oc_ (mV)	FF(%)	Efficiency (%)	Rs(Ω/cm^2^)	Rsh(Ω/cm^2^)
MoO_3_/Au	Front-Side	25.1	755	52.1	9.97	9.97	303.6
MoO_3_/Au annealing	Front-Side	25.6	752	57.1	11	7.69	332.2
MoO_3_/Au annealing	Back-Side	21	749	55.1	8.67	10.1	344
